# Rapidly Growing Squamous Cell Carcinoma of the Tongue

**DOI:** 10.7759/cureus.7164

**Published:** 2020-03-02

**Authors:** Dominick Myers, Emily Allen, Amr Essa, Maryam Gbadamosi-Akindele

**Affiliations:** 1 Internal Medicine, Creighton University School of Medicine, Omaha, USA

**Keywords:** oral cancers, tongue diseases, squamous cell carcinoma

## Abstract

The tongue can be a guide for different normal findings/variants, pathological lesions, or signs for systemic diseases. And oral cancer can be among the differentials, which can be detected early by a thorough oral exam. The early detection of oral cancer is particularly important, as the stage of oral cancer at the time of the diagnosis is the most critical factor determining the five-year survival. We present a case of a high-risk patient for oral cancers presented with a rapidly growing tongue lesion over six months that was diagnosed as a squamous cell carcinoma of the tongue on the biopsy. The case highlights an alarming rapid growth nature of oral cancers and alerts the clinicians of the importance of the physical exam as a cost-effective and potentially life-saving measure against oral cancers. It also demonstrates a brief review of risk factors and high-risk features of oral cancers.

## Introduction

Oral cancers represent 3% of total cancer incidence in the United States, of which squamous cell carcinoma (SCC) is the most common one representing over 90%. While the prognosis of many cancers has improved with medical advancements in recent years, the prognosis for oral cancers has not significantly changed [1]. A thorough periodic oral exam, especially in high-risk individuals and with precancerous lesions, is very cost-effective and can be life-saving as the stage of oral cancer at the time of the diagnosis is the most critical factor determining the five-year survival [1].

## Case presentation

**Figure 1:**
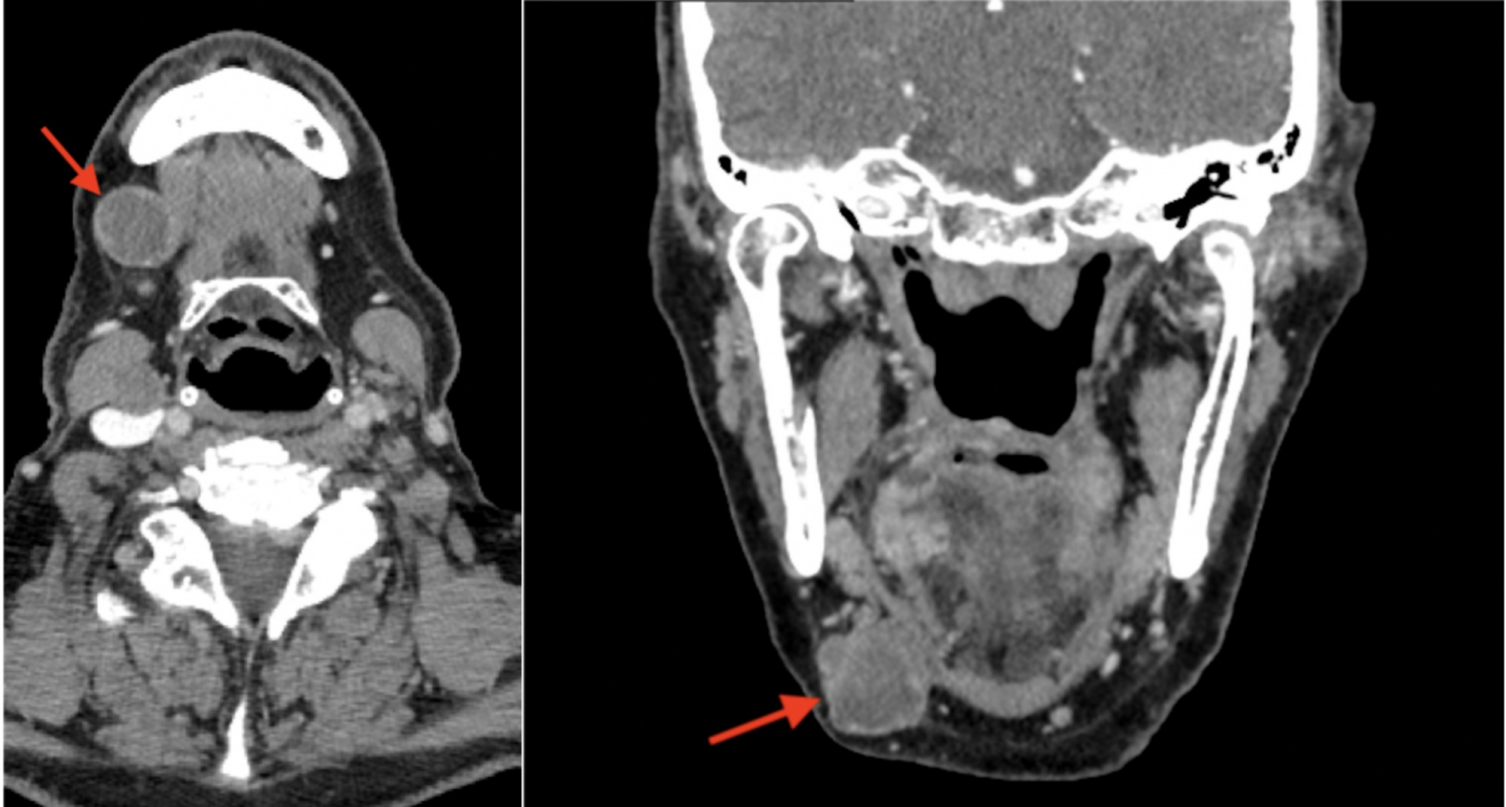
Head and neck CT showing hypodense rim enhancing soft tissue lesion in the right submandibular space (red arrows).

**Figure 2:**
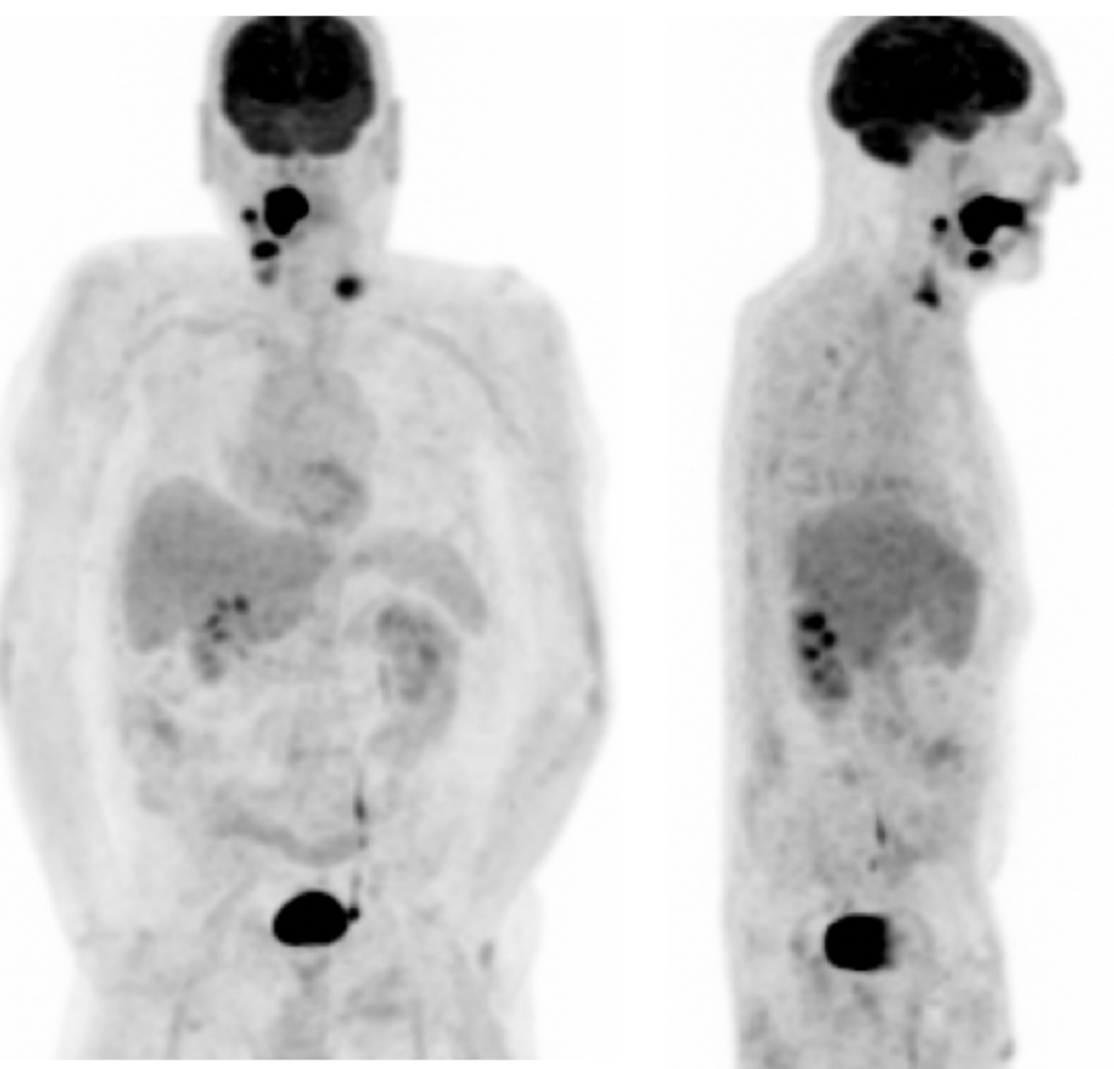
Whole body PET/CT scan showing increased metabolism in the tongue and nearby locations along with supraclavicular lymph node.

**Figure 3:**
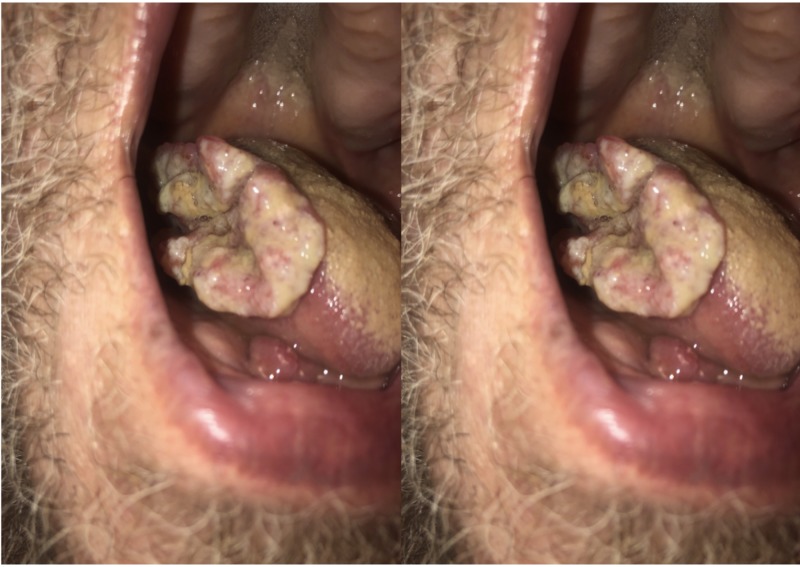
Exophytic, fungating, and indurated mass on the lateral tongue resembles squamous cell carcinoma (SCC) of the tongue.

A 62-year-old male presented to the clinic with a 2 x 5 cm lesion on the right lateral tongue that was described as erythroplakia with ulceration. The lesion first appeared approximately six months ago after he had bitten his tongue during a fall and had been growing since then. He had a 50-pack year smoking history and consumed vodka daily. A punch biopsy revealed a squamous cell carcinoma, and he had a computed tomography (CT) scan of the head and neck (Figure 1) along with a total-body positron emission tomography (PET)/CT scan (Figure 2) for staging which was CT4aN1. He was scheduled for surgery that was delayed because of unforeseen circumstances on the patient’s side. During his pre-operative evaluation, three months later, the lesion had been grown and measured 3 x 6 cm (Figure 3). He had a subtotal glossectomy with a tracheostomy, bilateral neck dissection, and a radial forearm free flap. His pathology revealed perineural invasion and extracapsular extension. He was started on cetuximab plus radiation therapy regimen.

## Discussion

Oral cancers can develop de novo, or from oral potentially malignant disorders (OPMDs) with leukoplakia and erythroplakia being the most common ones with a risk of malignant transformation of as high as 18% and 50%, respectively [2]. Tobacco and smoking are the most common modifiable risk factors, and 80% of patients with oral cancers had a smoking history [1]. Also, drinking four or more alcohol drinks per day has a 9.29% relative risk for developing oral cancer [3]. Further, the use of both of them concomitantly increases the risk by 100-fold in women and 38 in men [4]. Other risk factors have been identified, such as, immunosuppression, longstanding inflammation, lichen planus, human papilloma virus, and human immunodeficiency virus infection [5].

Our patient demonstrated a high-risk individual for developing oral cancer given his history of concomitant smoking and alcohol use, so a high index of suspicion should be warranted in evaluating such individuals. Also, his initial lesion of the lateral tongue might not have been readily visible on the oral exam; therefore in-depth oral exam is prompted as lesions can be obscured by oral structures and many oral cancer sites might not be visible on the initial exam, for example, the tongue posterior lateral and ventral surface which are the most common site for oral SCC [6]. So, during the evaluation of oral cavity for any lesions, any change in color, texture, or contour of the oral cavity should raise suspicion and a good way to remember the characteristics of high-risk lesions that might need a biopsy is any single mucosal lesion which is red and/or white, ulcer, lump or exceeding three weeks duration (RULE) [7].

Lastly, it is essential to understand the phenomenon of field cancerization in the development of oral cancer and precancerous lesions. Fields of altered cells can develop secondary to genetic mutations and long-term exposure of carcinogens. Those fields of cells can have normal morphology while the mucosa has acquired genetic alternations allowing malignant transformation. So, with more understanding of this phenomenon, it is vital to advise towards modifiable risk factor cessation, a thorough examination of the oral cavity and not only the morphologically abnormal lesion, and long-term follow-up as those fields can take few years to transform into cancer [8,9].

## Conclusions

Oral cancer lesions can be often asymptomatic until they are advanced, and the progression can occur rapidly. And regular, long-term screening for intraoral lesions in patients who are at high risk for oral cancer is a simple, cost-effective, and potentially life-saving preventative measure.
